# At the mercy of viruses

**DOI:** 10.7554/eLife.16758

**Published:** 2016-05-17

**Authors:** Claus O Wilke, Sara L Sawyer

**Affiliations:** 1 Department of Integrative Biology, The University of Texas at Austin, Austin, United States; 2 Department of Molecular, Cellular, and Developmental Biology, University of Colorado Boulder, Boulder, United Statesssawyer@colorado.edu

**Keywords:** adaptive evolution, human evolution, host/pathogen interactions, viruses, mammals, Human, Other

## Abstract

Viruses are responsible for many of the adaptive mutations in the human genome.

**Related research article** Enard D, Cai L, Gwennap C, Petrov DA. 2016. Viruses are a dominant driver of protein adaptation in mammals. *eLife* **5**:e12469. doi: 10.7554/eLife.12469

**Image** Many adaptive mutations (red) in proteins occur on or near binding sites for viruses (green)

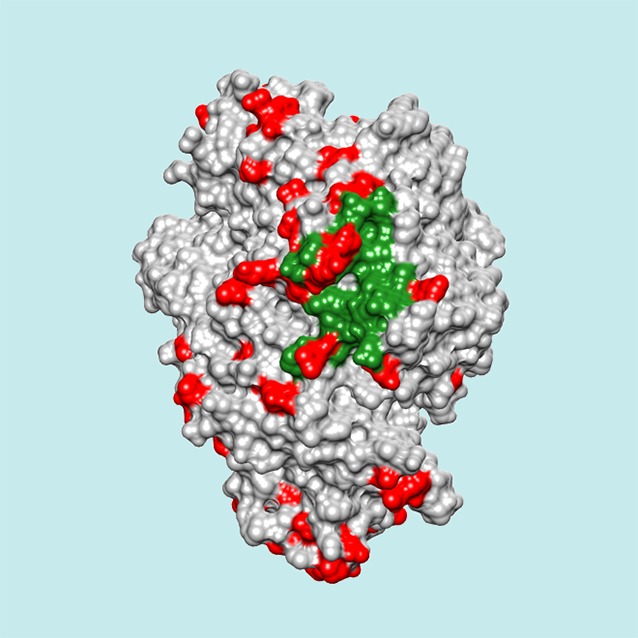

A long-standing quest in evolutionary biology has been to identify the mechanisms that cause genomes to change and diversify over time. One celebrated theory, the neutral theory, argues that the vast majority of mutations found in genomes are of no consequence, and that mutations persist entirely by random chance (Kimura, 1983). Alternatively, mutations may persist because they are adaptive; that is, the organism is better off having the mutation than not. Now, in eLife, David Enard, Le Cai, Carina Gwennap and Dmitri Petrov from Stanford University argue that viruses are one of the major drivers of adaptive mutations in the human genome and in mammalian genomes in general (Enard et al., 2016).

Enard et al. examined patterns of molecular evolution in human proteins, comparing the patterns in proteins that interact with viruses to those that do not. How do we know which human proteins interact with viruses? Since this information is not readily available, Enard et al. had to manually search the literature and read thousands of articles to build their data set. They started out with a list of 9,861 human proteins and then searched for all articles that mentioned one of the proteins as well as the word “virus.” Each article was then examined to determine whether it established a physical interaction between the human protein and either a viral protein, viral RNA or viral DNA. This procedure yielded a final list of 1,256 proteins that physically interact with viruses. The remaining proteins in the list were used as a control group, serving as point of comparison.

To assess the extent of adaptation in these proteins, Enard et al. used a test that compares patterns of mutations in the DNA sequences that encode the proteins (McDonald and Kreitman, 1991). This test separates mutations into four different types. First, it defines substitutions as those shared by all individuals in a population, and polymorphisms as those only present in some individuals. Furthermore, mutations can occur at “functional” sites, where they can modify the protein that gets expressed, or at “neutral” sites, where they have no effect on the final protein. Under the neutral theory, we expect the ratio of functional versus neutral mutations to be the same whether we are considering substitutions or polymorphisms (Figure 1A). Conversely, if some functional mutations are adaptive and result in increased fitness, then natural selection will have acted to drive an increase in the frequency of functional mutations until they are shared by all individuals. In this case, the ratio of functional versus neutral mutations will be *greater* for substitutions than it will be for polymorphisms (Figure 1B). More importantly for the study by Enard et al., the two ratios can also be used to estimate the fraction of substitutions driven by adaptation (Smith and Eyre-Walker, 2002).

**Figure 1.**
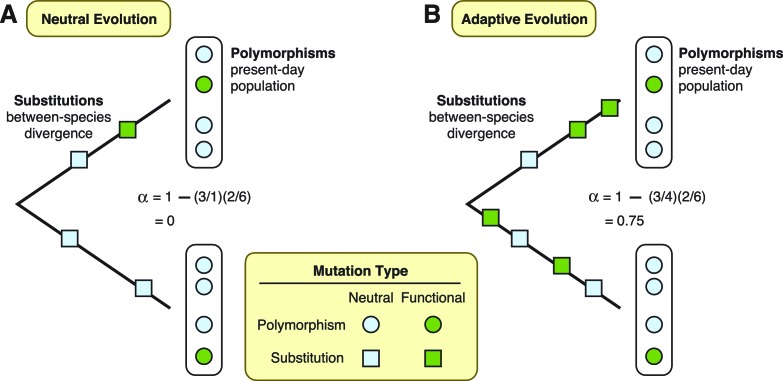
Two theories are diagrammed, neutral evolution and adaptive evolution. These theories describe how genetic mutations persist in the genome, leading to the development of new traits in a species. To differentiate between these two theories, the McDonald–Kreitman approach estimates the amount of adaptive evolution in diverging populations. This method involves comparing two ratios: *d/d*_0_, which is the number of substitutions at functional sites in a protein’s DNA sequence (*d*; green squares) divided by the number of substitutions at neutral sites (*d*_0_; light blue squares); and *p/p*_0_, which is the number of polymorphisms at functional sites (*p*; green circles) divided by the number of polymorphisms at neutral sites (*p*_0_; light blue circles). These two ratios can also be used to estimate the fraction (*α*) of substitutions driven by adaptation, via the formula *α *= 1 – (*d*_0_/*d)(p/p*_0_). (**A**) Under neutral evolution, we assume that all mutations at neutral sites have no effect on how the resulting protein works and that all mutations at functional sites either have no effect or are strongly deleterious. In this case, *d/d*_0_ = *p/p*_0_, and α = 0. (**B**) Under adaptive evolution, there is an excess of substitutions at functional sites relative to the number of polymorphisms seen at functional sites, i.e., *d/d*_0_ > *p/p*_0_. In this case, *α* > 0.

Enard et al. find that at least 30% of the adaptive mutations that have accumulated in human genes seem to have arisen because they offer protection against viral infection. This conclusion is based on the fact that more adaptive substitutions were found in the subset of proteins that interact with viruses than in the control set of proteins that do not. The fact that so much human evolution has been focused on proteins that interact with viruses affirms what we already knew: viruses have historically been one of our biggest causes of death and disease, if not the biggest cause. They, not lions, tigers or bears, sit masterfully above us on the food chain of life, occupying a role as alpha predators who prey on everything and are preyed upon by nothing.

One seemingly puzzling result from the study is that the human proteins that interact with viruses are more evolutionarily conserved than other proteins, yet also experience more adaptive evolution. However, this can be explained by considering where the adaptive mutations occur. Viruses may evolve to interact with the conserved and essential proteins of their hosts because targeting those proteins makes it easier for the viruses to infect all individuals within a species. This may also help the viruses to infect individuals of a new species. But unfortunately for the viruses, interacting with a host protein may promote adaptive mutations at the virus-binding surface of the protein, eventually preventing the viral interaction. This selection pressure creates a hotspot of adaptive evolution in a protein that is otherwise highly conserved.

The work by Enard et al. is conservative; it likely underestimates the total amount of selection due to viruses. As the authors point out, their list of virus-interacting proteins is only a subset of the actual virus-interacting proteins encoded by the human genome. We typically only know that a protein interacts with a virus if the virus causes a significant disease burden and is well-studied, such as HIV or influenza virus. There are, however, many other unknown viruses that we encounter every day through processes such as eating and breathing. An extensive network of proteins protects us from these viruses, but the proteins in this network are difficult to identify because they act silently, protecting us so successfully that no disease results. New human viruses such as Ebola, Zika and MERS arise when one of these environmental viruses evolves to circumvent this invisible shield. In this way, diverse human proteins are engaged in a silent battle with viruses every single day.
